# Massively parallel reporter assays discover de novo exonic splicing mutants in paralogs of Autism genes

**DOI:** 10.1371/journal.pgen.1009884

**Published:** 2022-01-20

**Authors:** Christy L. Rhine, Christopher Neil, Jing Wang, Samantha Maguire, Luke Buerer, Mitchell Salomon, Ijeoma C. Meremikwu, Juliana Kim, Natasha T. Strande, William G. Fairbrother

**Affiliations:** 1 Molecular Biology, Cell Biology and Biochemistry, Brown University, Providence, Rhode Island, United States of America; 2 Autism & Developmental Medicine Institute, and Genomic Medicine Institute, Geisinger, Danville, Pennsylvania, United States of America; 3 C enter for Computational Molecular Biology, Brown University, Providence, Rhode Island, United States of America; 4 Hassenfeld Child Health Innovation Institute of Brown University, Providence, Rhode Island, United States of America; HudsonAlpha Institute for Biotechnology, UNITED STATES

## Abstract

To determine the contribution of defective splicing in Autism Spectrum Disorders (ASD), the most common neurodevelopmental disorder, a high throughput Massively Parallel Splicing Assay (MaPSY) was employed and identified 42 exonic splicing mutants out of 725 coding *de novo* variants discovered in the sequencing of ASD families. A redesign of the minigene constructs in MaPSY revealed that upstream exons with strong 5’ splice sites increase the magnitude of skipping phenotypes observed in downstream exons. Select hits were validated by RT-PCR and amplicon sequencing in patient cell lines. Exonic splicing mutants were enriched in probands relative to unaffected siblings -especially synonymous variants (7.5% vs 3.5%, respectively). Of the 26 genes disrupted by exonic splicing mutations, 6 were in known ASD genes and 3 were in paralogs of known ASD genes. Of particular interest was a synonymous variant in TNRC6C - an ASD gene paralog with interactions with other ASD genes. Clinical records of 3 ASD patients with TNRC6C variant revealed respiratory issues consistent with phenotypes observed in TNRC6 depleted mice. Overall, this study highlights the need for splicing analysis in determining variant pathogenicity, especially as it relates to ASD.

## Author summary

Autism Spectrum Disorders (ASD) is the most common neurodevelopmental disorder that presents with both phenotypic and genetic heterogeneity. Before genetically defined therapeutic approaches can be applied to the treatment of ASD, it is crucial to understand the genetic basis of the disorder. Tremendous effort by consortiums, such as the Simon Foundation for Autism Research Initiative, has led to the identification of variants and, in turn, genes associated with ASD. However, the effect of ASD variants have on the process of splicing is often not fully considered when assessing ASD risk genes. Using a high-throughput splicing assay, MaPSy, ~6.3% of ASD-associated variants affected splicing, highlighting a substantial contribution of defective splicing to ASD. This analysis led to identification of genes, such as TNRC6C, that warrant further investigation with respect to ASD risk.

## Introduction

The remarkable advent of next generation sequencing technologies in the past decade has led to the discovery that each individual carries millions of genetic variants, including more than ~10,000 peptide-altering variants [1,2]. The challenge now lies in the ability to interpret and identify causal disease variants from thousands of potential functional variants. Classical variant interpretation methods rely heavily on the variant’s impact on the peptide sequence and its evolutionary constraint [3–5]. For example, nonsense, frameshift, and splice site mutations may lead to loss-of-function of the target gene and are expected to be deleterious (also known as likely gene disrupting). However, these methods cannot easily determine whether an exonic variant residing outside the canonical splice site may impact splicing thereby having a more deleterious effect than expected. Auxiliary splicing elements can be found throughout both the exonic and intronic sequences and add an additional level of regulation important in influencing splicing outcome. More specifically, exonic splicing enhancer motifs (ESEs) tend to enhance splicing by recruiting their corresponding trans-acting factors to aid in spliceosomal recognition. The disruption of ESE elements can result in reduced interactions with the core spliceosomal machinery and lead to aberrant and deleterious gene products. Recently we have developed a high-throughput reporter assay, MaPSy, to screen ~5,000 disease-associated variants as a functional approach to detect exonic splice altering variants on a high-throughput scale [6]. MaPSy revealed ~10% of exonic disease-causing variants disrupted splicing, highlighting the relevance of splicing in disease.

Autism Spectrum disorder (ASD) is the most prevalent neurodevelopmental disorder that is characterized by impaired communication and social skills, repetitive behavioral patterns, and restrictive interests. Although there is a high level of phenotypic and genetic heterogeneity associated with ASD, there remains a strong genetic component, with an estimated heritability of 40–90% [7–11]. As sequencing technologies are becoming more commonplace, the number of ASD-associated variants is increasing. These studies have identified many ‘candidate ASD genes’ by finding rare sequence variants and copy number variants (CNVs), many of which are *de novo*, that substantially contribute to ASD risk [12–15]. However, the individual contribution of these ‘candidate ASD genes’ to disease pathogenesis may be small, largely due to ASD’s phenotypic and genetic heterogeneity. Although great progress has been made in identifying ‘high confidence candidate genes’ via the recurrence of likely gene-disrupting variants, these studies often overlook the potential gene-disrupting role of non-canonical splicing [16–18]. For example, a previously classified synonymous or missense variant could also have a large negative impact on a protein through its disruption of auxiliary *cis*-sequence motifs crucial for splicing. To investigate the contribution of defective splicing in ASD pathogenesis, *de novo* variants identified in ASD participants enrolled in the Simons Simplex Collection study (SSC, i.e. ~2,500 ASD families [16]) were evaluated with our high-throughput splicing assay [6] for their effect on splicing outcome. The assay revealed that 6.3% of *de novo* coding variants seen in individuals with ASD significantly altered splicing and warrant further investigation. We identified 7 splice altering variants in paralogous genes, which may suggest that paralogs of genetic risk genes can themselves be genetic risk factors. For example, we identified a splice altering variant in *TNRC6C*, *a paralog of the SSC* ‘high confidence candidate gene’ *TNRC6B* [16,19–24]. We report potential involvement of *TNRC6C* in ASD pathogenesis, especially as it pertains to the respiratory issues faced by the ASD children with mutations in *TNRC6C* and its paralogous genes, *TNRC6B* and *TNRC6A*. Furthermore, an analysis on pathways associated with ASD revealed new potential ASD candidate genes that warrant further investigation with respect to ASD risk.

## Results

### Flanking splice site strength influences level of variant perturbation

**Fig 1:**
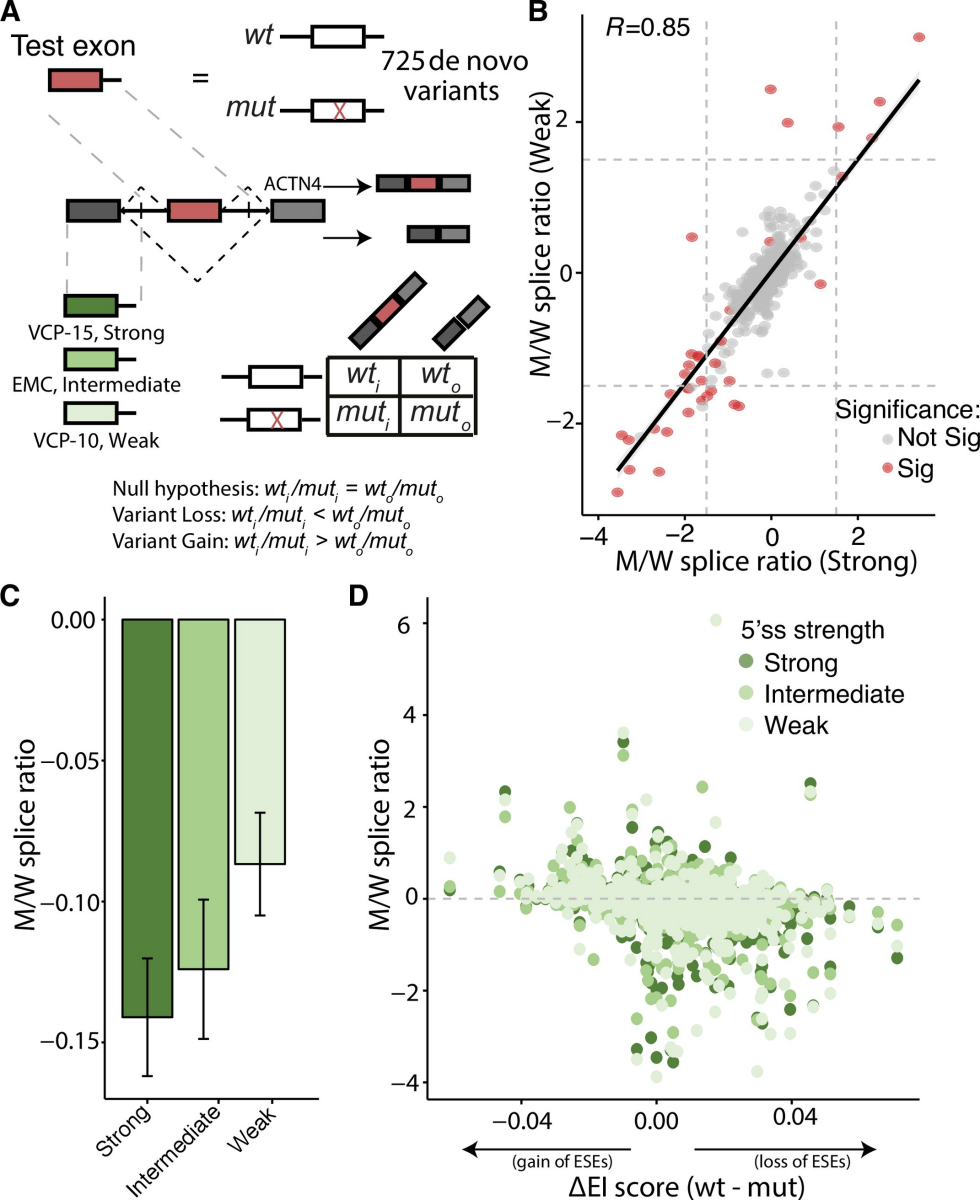
Preceding 5’ss strength influences level of variant perturbation. **A.** A total of 725 coding *de novo* coding variant exons and their corresponding wild type counterparts were incorporated into three different three-exon *in vivo* constructs. Both the input and spliced output libraries were deep sequenced to establish allelic mutant and wildtype ratios. **B.** Comparison of individual mutant and wild type allelic ratios in the VCP exon 15 and VCP exon 10 minigene reporter constructs. Red points indicate significance in one, two, or all three of the minigene reporters (see **S1 Fig** for additional comparisons). **C.** Mean allelic imbalance of all coding *de novo* variants in each minigene construct (error bars represent s.e.m.) **D.** Comparison of a variant’s allelic imbalance to the change in splicing enrichment index (32) (i.e. wild type EI score–variant EI score). A positive value indicates the loss of ESEs (right) and a negative value indicates the gain of ESEs (left).

While massively parallel reporter assays (MPRA) offer an efficient way to screen thousands of disease variants, minigenes are not physiological as they often test splicing effects in chimeric constructs that differ from the original transcript. To test the validity of the MaPSy minigene reporter approach in accurately assessing the impact on splicing, 725 coding *de novo* variants discovered in ASD families sequenced as part of the SSC study [16] were screened in three separate minigene reporters. The previously described [6] minigene construct was modified to introduce three different reporter exons with varying 5’splice site (5’ss) strengths. Each reporter contained either a strong (VCP exon 15, MaxEntScan = 10.15), an intermediate (EMC7 exon 3, MaxEnt = 8.35), or weak (VCP exon 10, MaxEntScan = 6.66) first exon, in addition to a 230-mer genomic sequence corresponding to either the mutant (M) or wild type (W) exon, and a downstream *ACNT4* exon (**Fig 1A**). Despite the differences in the reporter constructs, general agreements were observed between the allelic imbalances (i.e. M/W splice ratios) in all three minigene constructs (**Figs 1B and** S1), confirming the reliability of the MaPSy screening approach.

While the rank order of aberrant splicing is maintained across different minigene reporters, the effect size is not. When the variants were assayed in the three-exon construct with the strongest first-exon 5’ss, the variants resulted in a greater allelic imbalance, indicative of more exon skipping events, as opposed to the intermediate and weak 5’ss reporters (**Fig 1C**, Kruskall-Wallis *P =* 0.031). Mechanistically, this suggests that splicing favors exon skipping when a variant exon is preceded by an exon with a strong 5’ss. This phenomenon offers an interesting avenue for potential therapeutic intervention. The weakening of an upstream exon could be implemented to restore the aberrant splicing of a downstream exon.

To mechanistically determine a variant’s mode of splicing disruption, the variant’s effect on auxiliary *cis*-sequence splicing elements were considered. The empirically determined enhancer activity score (EI) [25–26], a metric corresponding to the enhancing and silencing effect of all possible hexamers in several exonic positions in multiple minigene substrates, were associated with each wild type and variant exon. As expected from the minigene results, the change in EI score of wild type and variant exons revealed that the loss of ESE elements correlated with a greater degree of allelic imbalance (i.e. more exon skipping events, **Fig 1D**). Thus, the majority of the apparent splicing defects are due to the loss of essential splicing signals.

### 6.3% of ASD-associated de novo coding variants disrupt splicing

**Fig 2:**
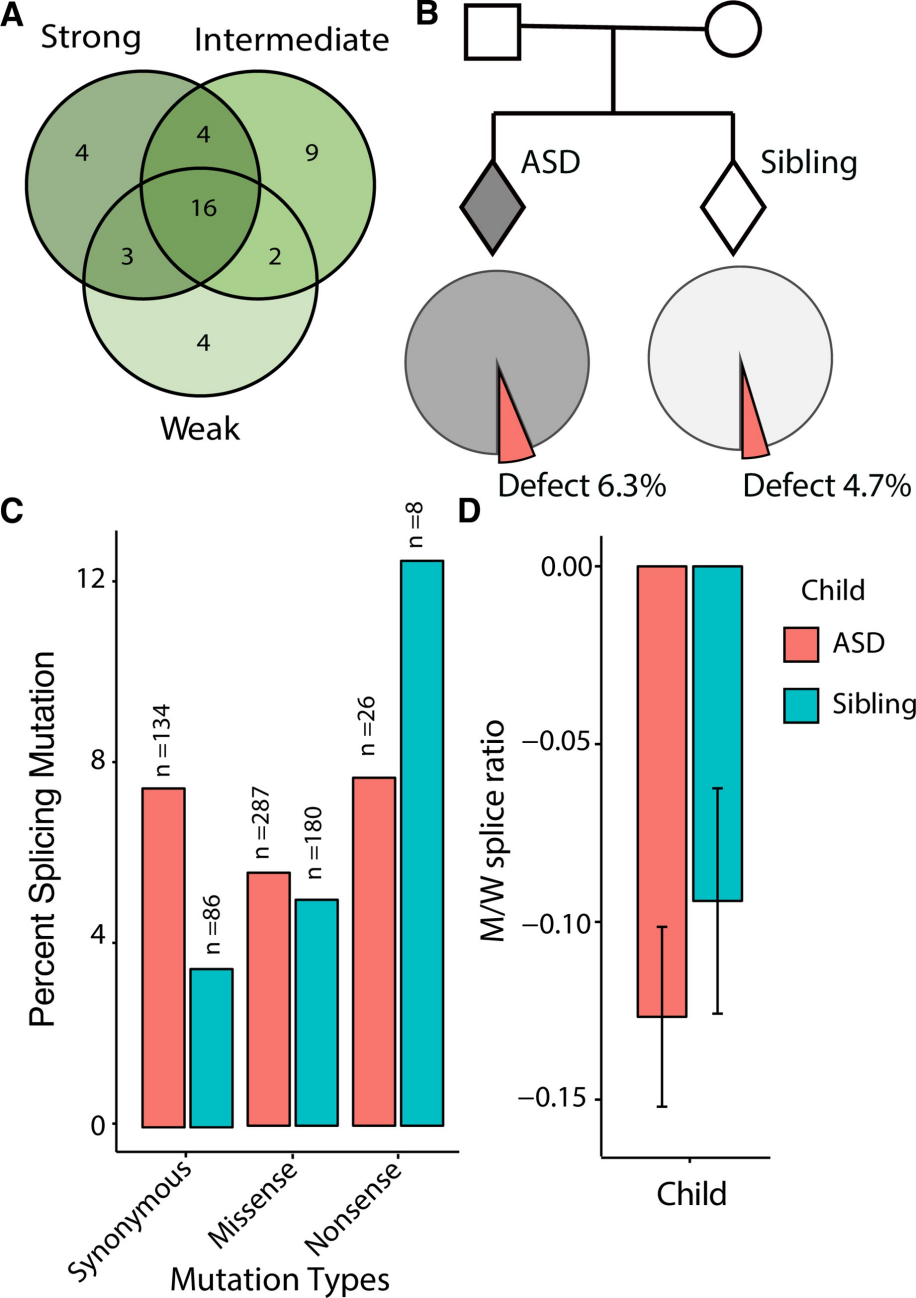
*De novo* synonymous variants disrupt splicing. **A.** Number of variants that significantly disrupted splicing in each minigene reporter construct. **B.** Example pedigree depicting an affected (gray diamond) and unaffected sibling (white diamond). Diamonds represent either male or female. Of the 447 assayed *de novo* variants observed in those with ASD, 6.3% significantly disrupted splicing (n = 28). Of the 274 assayed *de novo* variants seen in unaffected siblings, 4.7% disrupted splicing (n = 13). **C.** Proportion of screened synonymous, missense, and nonsense variants that disrupted splicing in ASD probands (red) and unaffected siblings (teal). **D.** Mean allelic imbalance (averaged M/W splice ratios across minigene reporters) of synonymous, missense, and nonsense variants in ASD probands (red) and unaffected siblings (teal) (*p*-values represent Mann-Whitney U test and error bar represent s.e.m).

A primary goal of this study is to discover *de novo* variants that increase risk for developing autism. A total of 42 of the coding *de novo* variants discovered in the autism cohort significantly disrupted splicing either in one, two, or all of the minigene constructs (**Fig 2A**: ≥ 1.5-fold change, two-sided Fisher’s exact test, adjusted with a 5% false discovery rate). Overall, 5.8% of coding *de novo* variants disrupted splicing. The *de novo* variants assayed can be further divided into those observed in an ASD child (447 variants) or unaffected sibling (274 variants). Further categorization of variants discovered in ASD families revealed a higher proportion of *de novo* variants in the ASD cohort disrupted splicing compared to *de novo* variants in their unaffected siblings (6.3% vs 4.7%, respectively, **Fig 2B**). Of the 42 *de novo* variants that alter splicing, 14 are synonymous and 25 are missense variants. Although there was a higher proportion of missense variants that affect splicing in ASD children (5.6% and 5.0% in ASD children and siblings, respectively), the contribution of splicing variants was even more pronounced when considering synonymous variants. Of the 14 *de novo* synonymous MaPSy detected splicing variants, 10 were reported in ASD children and 3 in unaffected siblings. Overall, 7.4% of the synonymous variants in ASD children disrupt splicing, compared to only 3.5% of synonymous variants in unaffected siblings (**Fig 2C**). Therefore, *de novo* variants seen ASD patients that seemingly lack a direct on the protein (i.e. synonymous variants) may still result in a disrupted peptide via perturbation of splicing.

### New ‘candidate ASD genes’: TNRC6C

**Fig 3:**
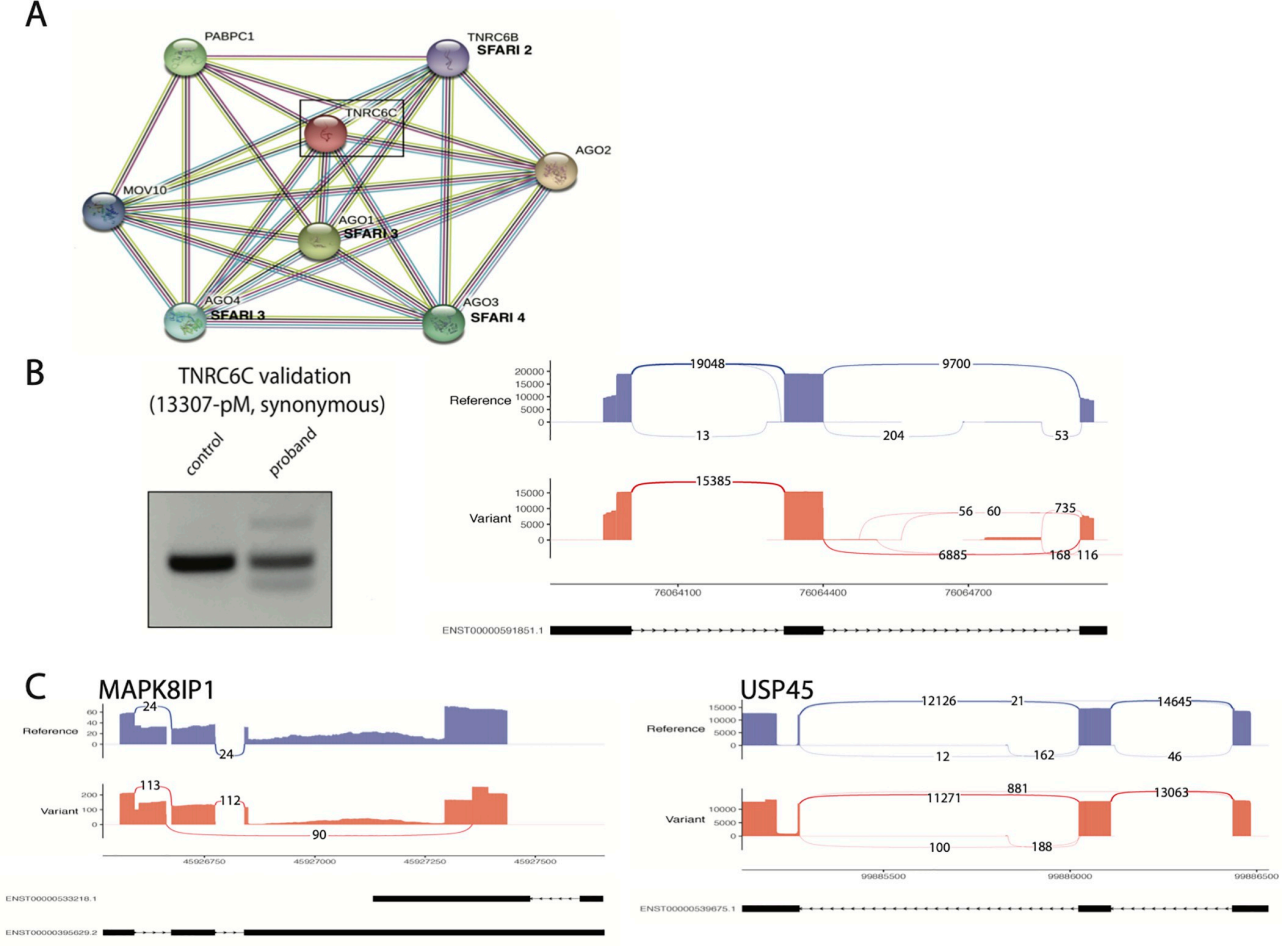
Protein interaction network of TNRC6C reveals connections to ‘candidate ASD genes’. **A**. STRING interaction network of TNRC6C highlights interactions with genes associated with ASD risk. **B.** Tissue sample validation of TNRC6C (left) shows upregulation of alternative splicing events in the proband sample, particularly as skipping event (88 bp band). Amplicon sequencing sashimi plot (right) confirms complex splicing phenotype in proband (variant) relative to control sample (reference). **C**. Amplicon sequencing Sashimi plots (left) display complex splicing phenotypes, particularly exon skipping, in the variant sample relative to the control for MAPK8IP1 and a less substantial splicing phenotype of the *USP45* variant relative.

Of the 26 genes with *de novo* splice altering missense and synonymous variants in ASD probands, 6 were previously reported in Simons Foundation for Autism Research Initiative Gene database (SFARI Gene) as having an association with ASD (*CHKB*, *CACNA2D1*, *ERBB2IP*, *SYNGAP1*, *TCF4*, *USP45*, S2 Table). Of the remaining 20 genes, 3 genes have paralogs reported as ASD-associated in SFARI Gene: *TNRC6C*, *MAPK8IP1*, and *HACE1* (S2 Table). The STRING database was used to dissect the interaction network of *TNRC6C* which revealed multiple direct interactions with genes reported as ASD-associated genes in SFARI (*AGO1*, *AGO3*, and *AGO4*, **Fig 3A**), strengthening the case for the involvement of *TNRC6C* in ASD pathogenesis. The Argonaute (AGO) proteins recruit TNRC6 proteins to bind to miRNAs by specific N-terminal interactions, forming an miRNA-mediated decay complex. Both AGO and TNRC proteins are required for RNA mediated gene silencing and have similarly short ribosomal binding half-lives. Specifically, TNRC6C has two unique motifs AGO binding interactions that are necessary for AGO’s translational repression of mRNAs [27–28]. This data is supportive of a role for these genes in ASD pathogenesis and may allow some to be reprioritized as high confidence ASD candidate genes and others classified as possible new ASD candidate genes.

Given the association between *TNRC6C*, *MAPK8IP1*, and *HACE1* with SFARI genes and the identification of the splicing variants in previously classified SFARI genes, patient validations using RT-PCR were performed to test for splicing defects in patient cell lines from the SSC (**Figs 3B, 3C and** S2). A clear difference in the splicing phenotype of *TNRC6C* between the parental control and variant cell lines was apparent (**Fig 3B, right**). However, the splicing event was complex. Minigene constructs do not fully capture the endogenous sequence context surrounding a variant exon of interest due to the common flanking exons used for all variants screened. Although an exon skipping event was detected in the MaPSy assay, it is not surprising that splicing event is more complex in the variant’s endogenous sequence. Amplicon sequencing was performed to identify the precise complex splicing phenotypes seen in the RT-PCR assay (**Fig 3B, left**). Additional, patient cell line validations were also executed for the splicing variants detected in *MAPK8IP1*, *HACE1* and the 6 SFARI genes (S2 Fig). Aberrant mis-splicing events were apparent for ASD paralog *MAPK8IP1*, and to a lesser and complex degree in the SFARI gene, *USP45* in the patient cell lines compared to their parental control, as seen in the amplicon sequencing results (**Fig 3C, left and right respectively**). Aberrant splicing events were also detected in patient vs control cell lines in 4 of the 6 additional SFARI genes (S2A Fig) and in the SFARI paralog, *HACE1* (S2B Fig).

### Post synaptic density protein genes are sensitive to splicing disruption in autistic children

**Fig 4:**
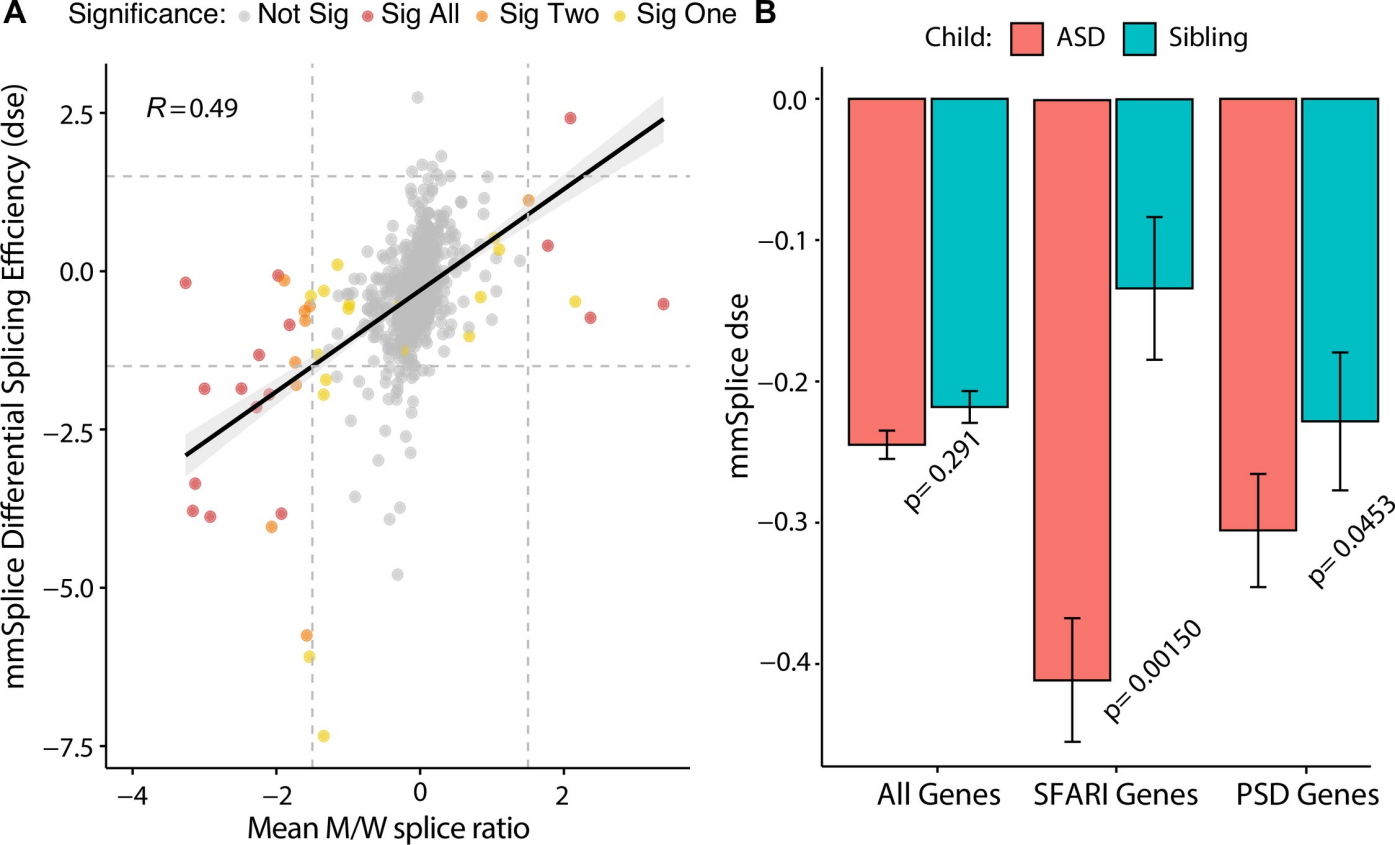
Splicing predictions reveal PSD gene de novo variants in ASD probands commonly disrupt splicing. Relationship of mmSplice predicted differential splicing efficiency (dse) to experimentally determined allelic imbalance. Dashed lines represent 1.5-fold change cutoffs for calling a significant splicing defect. Labels indicate constructs where significance was established for each variant. **B.** Left, mean mmSplice predicted dse of *de novo* coding variants associated in all genes (left), SFARI category 1–5 genes (middle), and post-synaptic density protein genes (right) in ASD children (red) vs unaffected siblings (teal). (*p*-values represent Mann-Whitney U test and error bar represent s.e.m).

To determine the overall contribution of defective splicing of variants in ASD probands, a further analysis of the allelic imbalance of variants in ASD probands and siblings revealed a greater allelic imbalance for variants in ASD probands as opposed to unaffected siblings, although not significantly (**Fig 2D**). Due to the current limitations of oligonucleotide synthesis, the lack of significance can in part be explained by the small sample of synonymous, missense, and nonsense *de novo* variants assayed (n = 725). In order to clearly estimate the role of splicing mis-regulation of *de novo* variants in ASD probands, a larger sample size would be required. The development of the splicing prediction models offer additional variant screening methods in determining the potential splicing disruption of possible ASD-associated variants [29]. Recently, a neural network prediction program (mmSplice) was trained on multiple splicing datasets (including the initial MaPSy data) to develop reliable splicing predictions given a variant [30]. One of the prediction modules of mmSplice is a differential splicing efficiency (dse) metric, which corresponds to the M/W splice ratio (also referred to as allelic imbalance) determined in the MaPSy assay. The comparison of the mmSplice predicted differential splicing efficiency, and the average allelic imbalance (across all three minigene reporters) of the 725 *de novo* variants suggests a strong correspondence between predicted and measured allelic imbalance, especially when variants were found to be significant in all three of the minigene reporters (**Fig 4A**, bottom left red points).

Given the correspondence between the mmSplice classifier and the measured allelic imbalance, the model was further used to screen the entire set of *de novo* synonymous, missense, and nonsense mutations reported in the *de novo* SSC dataset. *De novo* variants in ASD probands are predicted to be more disruptive to splicing as opposed to variants reported in unaffected siblings, as evident in the assay results (**Fig 4B, left bar**). Restricting the *de novo* variants to ASD-associated genes (as reported in the Simons Foundation for Autism Research Initiative Gene database), revealed that SFARI gene *de novo* variants in ASD probands led to a greater degree of allelic imbalance as opposed to unaffected siblings (**Fig 4B, middle bar,** Mann-Whitney U *P =* 1.50e-3).

In addition, previous reports have presented data suggesting the relevance of pathway analysis in assessing the role of variants discovered in ASD [12,16,31]. To determine the role of defective splicing of variants in the gene networks commonly associated with ASD, seven pathways were considered: fragile X mental retardation target (FMR1), chromatin modifiers, post-synaptic density protein genes, genes expressed during embryonic development, cell adhesion molecules, Calcium signaling, and Wnt-signaling. Of the pathways considered, *de novo* variants in ASD probands seen in post-synaptic density (PSD) genes displayed a significant increase in defective splicing as opposed to unaffected siblings (**Fig 4B, right bar,** Mann-Whitney U *P =* 0.04553), highlighting the contribution of defective splicing in PSD genes to ASD.

## Discussion

It is apparent by the MaPSy results for *de novo* variants in ASD probands that both synonymous and missense variants outside of the core splice-site signals can still have a deleterious effect on the overall splicing outcome of a transcript, but may be overlooked by classical variant interpretation methods. The MaPSy screen revealed that 6.3% of de novo variants in ASD probands led to a splicing defect and may warrant reclassification as ‘likely gene disrupting variants’ (**Fig 2**). Through screening the ASD variants in multiple minigene constructs with MaPSy, it became apparent that flanking splice-site strength influences the level of a variant’s perturbation on splicing, a phenomenon that can be exploited in therapeutic intervention. These ‘likely gene disrupting variants’ may aid in the identification of new ‘high confidence ASD candidate genes’ via the recurrence of likely gene-disrupting variants [14,16, 20,21,23,31–34].

Gene scores used in SFARI directly correlate to the quantity of evidence relating a gene to ASD and the categories provide an additional effective system of labelling the highest priority genes for reference in ASD genomic sequencing and clinical diagnostics. The collection of genetic evidence for ASD suggests future research directions for understanding the causes and mechanisms of autism. Therefore, there is a high importance in identifying new candidate genes. A pathway investigation of the 42 genes containing *de novo* splicing disrupting variants to determine interactions with ASD pathogenesis further suggests that *TNRC6C* may be a new ‘candidate ASD gene.’ The *TNRC6* family of proteins (GW182 –*TNRC6A*, *TNRC6B*, and *TNRC6C*) plays an important role in miRNA-dependent translational repression [35]. A recent study found *TNRC6C* knock-out mouse models resulted in respiratory failure and ultimately death within 24 hours of birth, highlighting its essential role in the developing lung. The extensive phenotypic data available for the SSC showed that the ASD child with a TNRC6C splice variant (17:76067246:G:A) appeared to suffer from respiratory issues [28]. In addition, the two children with likely gene-disrupting variants (as defined by SFARI) in *TNRC6C* were both diagnosed with respiratory issues. The TNRC6 protein family, which includes, TNRC6C also has multiple reports in the literature of overlapping interactions with FMR1, the gene implicated in fragile X syndrome [36–37], which is one of the seven pathways prioritized in our analysis of the *de novo* variants. To further confirm the MaPSy splicing results, patient cell line splicing validation was then performed on the synonymous *TNRC6C* variant with clear results indicating that the variant disrupted splicing in the endogenous environment (**Fig 3A**). These findings suggest that defects in the *TNRC6* family of genes may increase risk for developing autism and should be further investigated.

As more variants are identified in ASD patients, it is likely that a high proportion of the variants will also have an impact on splicing. It will be important to incorporate splicing analysis in determining ‘likely gene-disrupting’ variants to aid in the identification of recurrently disrupted genes associated with ASD. The results in this study highlight the importance of utilizing splice predictors such as mmSplice or minigene splicing reporter assays when interpreting the impact of variants.

## Methods

### In vivo splicing reporters

The 725 coding *de novo* variants within exons of ≤115 nucleotides were selected. Solid-phase oligonucleotide synthesis technology was used to generate a 230-mer oligonucleotide library of substrates corresponding to 180-mer nucleotide (nt) genomic fragments containing either mutant or wild type (reference) sequence exons, at least 50nt of the upstream intron, 15nt of the downstream exons, and 25 nt on either side of the oligo for primer sequences.

The three separate *in vivo* splicing reporters were generated using overlapping PCR and consists of the Cytomegalovirus (CMV) promotor, either *VCP* exon 15, *EMC* exon 7, or *VCP* exon 10 with part of their downstream introns at the 5’ end, followed by the 230-mer library, and exon 16 of *ACTN4* with part of intron 15 and the bGH polyA signal sequence at the 3’ end [38]. The resulting in vivo reporters were transfected into human embryonic kidney hek293T cells. After 24 hours of transfection, RNA was extracted and both the input reporters and spliced output were converted to cDNA, PCR amplified and deep sequenced.

### Library species alignment and counting

Alignment and counting was done as previously described [6]. A “reference genome” was created where each pair of wild-type and mutant species were treated as their own “chromosome”. STAR aligner was used to map paired-end reads. For the input library, split reads were discarded and only uniquely mapped reads were allowed. The same settings were used for the spliced output, with the exception of allowing for split reads.

### Allelic imbalance calculation

The allelic imbalance for MaPSy analyses were calculated as:

$$\frac{M}{W}splice\;ratio={Log}_{2}\left(\frac{m_{o}/m_{i}}{w_{o}/w_{i}}\right)$$

where *m_o_* is the count of mutant spliced species, *m_i_* is the count of mutant input, *w_o_* is the count of wild-type spliced species and *w_i_* is the count of wild-type input. To assess statistical significance, a two-sided Fisher’s exact test was used and the resulting P values were adjusted to account for multiple comparisons using the p.adjust function in R (method = `fdr’). A significance level of <0.05 and an allelic ratio of ≥1.5-fold were used to call significant splicing disruptive variants.

### MMSplice predictions

All synonymous, missense, and nonsense SSC *de novo* variants were assessed for their predicted effect on splicing using mmSplice [30] (n = 3935). Variants with multiple predicted scores were averaged to obtain a single predicted value per variant.

### Pathway analysis

The predictions for the SSC *de novo* variants were intersected with genes in 7 ASD associated pathways: calcium signaling, cell adhesion molecules, embryonically expressed, chromatin remodeling, essential genes, fragile X mental retardation target genes, post synaptic density protein, and Wnt Signaling genes. Chromatin modifier, embryonically expressed, essential genes, and fragile x mental retardation target genes were obtained from a previous ASD study [16]. Calcium signaling, cell adhesion molecule, and Wnt signaling genes were obtained from KEGG. The SFARI category 1–5 genes were download from SFARI gene on July 26^th^, 2019. Proband and sibling variants were separated and averaged to assess the contribution of defective splicing.

### Gain and loss of exonic splicing regulatory signals

All possible hexamers, at a step of one nucleotide, in each variant and wild type exonic sequence were associated with their corresponding EI score [25], a metric reflecting the ability of a sequence to enhance or silence splicing. The EI score was averaged over the length of the exonic sequence to reflect the relative enhancing ability of each wild type and variant exon. The change in EI score was calculated at the average wild type exon EI score minus the average variant exon EI score.

### Patient validation

Splicing variants found to significantly disrupt splicing in the minigene assay for *TNRC6C*, *MAPK8IP1*, and *USP45* were selected for validation in patient samples. Lymphocyte cell lines corresponding to the child containing the variant of interest and as a control, the corresponding mother’s cell line were obtained from the Simons Foundation. RNA was extracted using TRIzol (Life Technologies) using the manufacturers protocol. SuperScript III Reverse Transcriptase (Invitrogen) was used to generate cDNA, followed by PCR (GoTaq, Promega). PCR primers were designed in exons flanking the variant exon and were designed to anneal to regions present in all reported isoforms (UCSC Genome Browser Human hg19 genome). To prevent skewed results due to nonsense mediated decay, cells were also treated with 10μg/ml cycloheximide for 3 h before RNA extraction.

To analyze the amplicon sequencing, two genomes were constructed containing the sequences around the variants of interest (starting at the beginning of the exon upstream of the variant exon and ending at the end of the exon downstream of the variant exon). One genome is the reference hg19 sequence and the other is the reference sequence with the variant substituted. Reads from the sequencing of the maternal cell lines were mapped to the reference genome, and reads from the sequencing of the patient cell lines were mapped to the mutant genome with STAR (used options—outFilterMismatchNmax 3 and—twopassMode Basic). The resulting BAM files as well as the GENCODEv19 transcript annotation were then input to ggsashimi [39] to create Sashimi plots around each variant’s locus (used option -M 10 to limit the displayed splice junction to those that have at least 10 reads supporting them).

## Supporting information

S1 Fig Comparison of individual mutant and wild type allelic ratios in the VCP exon 15 and EMC7 (Left) and VCP exon 10 and EM7 (right) minigene reporter constructs. Red points indicate significance in one, two, or all three of the minigene reporters.
(TIFF)

S2 Fig RT-PCR Patient (left lane) vs. parental (right lane) cell line validations for 6 SFARI genes (*CHKB*, *CACNA2D1*, *ERBB2IP*, *SYNGAP1*, *TCF4*, *USP45)*, *MAPK8IP1* and *HACE1*). Bands labelled (right) with expected exon splicing. Higher weight bands indicate WT exon splicing events. Lower weight, aberrant splicing bands show expected exon skipping events in probands.
(TIFF)

S1 Table All de novo variants screened in the MaPSy assay, with the corresponding mutant vs wildtype splicing ratio detected in the assay. (XLS)

S2 Table De novo proband variants showing significant differences in splicing compared to wildtype with the SFARI gene category reported, if applicable, and if they were found to be paralogs of SFARI ‘ASD risk’ genes. (XLSX)
