# Supplementary figures and images for: Massively parallel reporter assays discover de novo exonic splicing mutants in paralogs of Autism genes
Source: PLoS Genet. 2022 Jan 20;18(1):e1009884. doi: 10.1371/journal.pgen.1009884 (PMC8775188; doi:10.1371/journal.pgen.1009884)

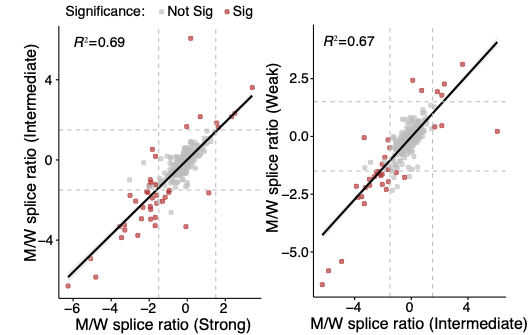

Supplement: S1 Fig — Red points indicate significance in one, two, or all three of the minigene reporters. (TIFF) [file pgen.1009884.s001.tiff]

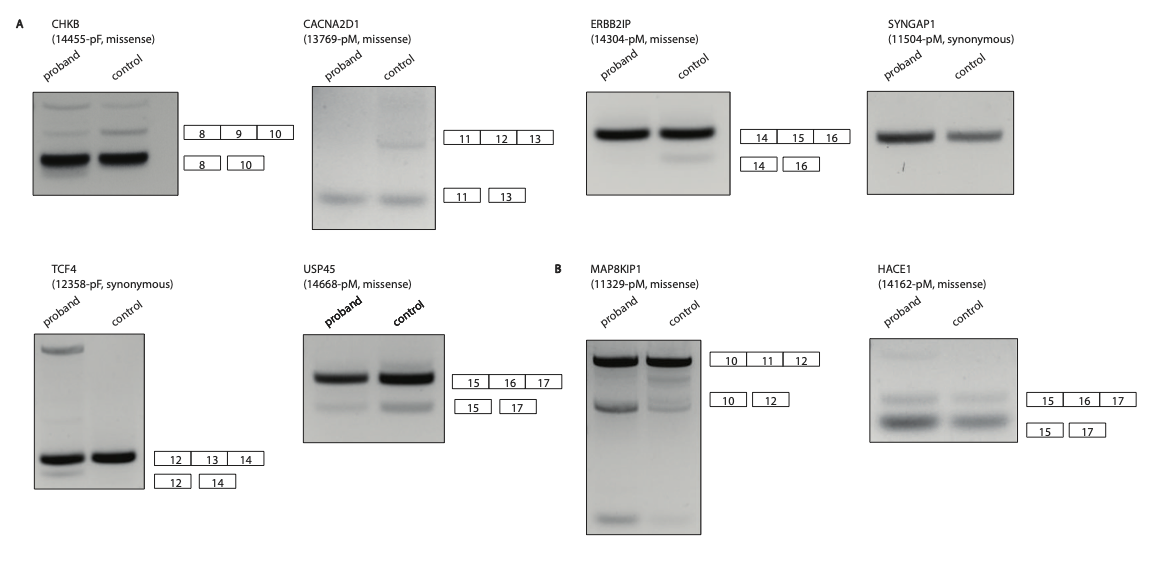

Supplement: S2 Fig — Bands labelled (right) with expected exon splicing. Higher weight bands indicate WT exon splicing events. Lower weight, aberrant splicing bands show expected exon skipping events in probands. (TIFF) [file pgen.1009884.s002.tiff]
